# Why did the human brain size evolve? A way forward

**DOI:** 10.1098/rstb.2024.0114

**Published:** 2025-06-26

**Authors:** Mauricio González-Forero, Aida Gómez-Robles

**Affiliations:** ^1^ Konrad Lorenz Institute for Evolution and Cognition Research, Klosterneuburg A-3400, Austria; ^2^ Department of Anthropology, University College London, London WC1H 0BW, UK

**Keywords:** human evolution, brain evolution, evo–devo, life history, mathematical model, simulation-based inference

## Abstract

Why the human brain size evolved has been a major evolutionary puzzle since Darwin, but addressing it has been challenging. A key reason is the lack of research tools to infer the causes of a unique event for which experiments are not possible. We suggest that analogous problems have been successfully addressed in other disciplines using what has been recently termed simulation-based inference. Following that approach, we outline a strategy to address why the human brain size evolved: hypotheses are expressed in mechanistic models that yield quantitative predictions for evolutionary and developmental trajectories of brain and body sizes, the predicted trajectories are compared with data, and models are chosen by their ability to explain the data. We discuss a recently published model that makes quantitative predictions for evolutionary and developmental trajectories of brain and body sizes for six hominin species, and compare the model predictions with data, finding that the model recovers many aspects of hominin evolution and development. Counter-intuitively, the human brain size evolves in this model as a spandrel or by-product of selection for something else, namely, fertility-determinant traits. Our analysis indicates that simulation-based inference offers a way forward to infer why the human brain size evolved.

This article is part of the Theo Murphy meeting issue ‘Selection shapes diverse animal minds’.

## 1. Introduction

Human evolution is characterized by a large brain expansion observed over the last 4 million years. Australopiths had a brain size about two times larger than expected for their body size, slightly larger than the brain size of chimpanzees. Over time, brain size tripled from australopiths to modern humans and became over five times larger than expected for the body size of *Homo sapiens* [1]. Evidence suggests that this brain expansion involved a concomitant increase in neuron number, as interspecific comparisons show that larger brains tend to have more neurons [2]. Increasing the number of neurons in artificial neural networks (typically implemented by increasing the number of parameters, or ‘synapses’) is key to increase their performance given a network architecture, and doing so has played a central role in the ongoing revolution of artificial intelligence [3,4, fig.22.7;^5^]. These and other lines of evidence suggest that hominin brain expansion underpinned large increases in cognitive abilities.

A longstanding question has thus been *why* such brain expansion happened. This is a ‘why’ question in that it asks for causes of a phenomenon, rather than a ‘how’ question that asks for descriptions of the phenomenon. This ‘why’ question is particularly challenging and has been thought to be unanswerable [6–9]. Indeed, while ‘why’ questions can be answered by studying the effects of interventions that are either human-made (as in artificial selection experiments) or natural (as in comparative analyses), both types of interventions are often infeasible for human brain expansion: human-made experiments are often infeasible because of practical or ethical reasons and natural experiments because the question of why human brain expansion occurred asks about the causes of patterns observed in a single lineage [10,11]. For instance, although studies in non-human animals enable experimental analyses that can assess causes [12,13], the causes of brain expansion may be lineage-specific, so what causes brain size evolution in other taxa, including non-human primates, may not necessarily be what caused human brain expansion. Consequently, the question asks about an effectively single data point corresponding to humans for which data must typically be observational, leaving the problem of why the human brain size evolved with a severe dearth of research tools.

An active approach to the question is based on correlational analyses. In that approach, hypotheses are formulated, which often emphasize ecological [14–17], social [18–21] or cultural [22–26] factors as selecting for larger brains. Then, proxy variables are chosen as being relevant to each hypothesis, such as diet type [27,28], environmental variability [29,30], group size [20,31] or social learning frequency [32], and these variables are tested with increasingly refined methods and larger datasets for whether the proxy variables correlate with brain size or the size of particular brain regions. Finally, if the proxy variable of a hypothesis correlates with the brain variable, or if it is more strongly correlated than the other proxy variables, then the correlations are interpreted as supporting the hypothesis. However, such a conclusion does not necessarily follow from the analysis, including because those correlations do not imply selection as they may arise from myriad other reasons, the directions of causality may be reversed to the given interpretation, causal connections between the variables may not be evolutionary, and correlations may be spurious [33–35]. For instance, if brain size correlates with environmental variability or group size within or among species, this might not be because environmental variability or larger groups caused larger brains to evolve, but because brains that evolved larger sizes for other reasons also enable subsistence under higher environmental variability or in larger groups. Hence, these correlational analyses provide limited evidence for or against the hypothesis considered.

We outline an alternative way forward to infer why the human brain size evolved, using an approach that has been pivotal across many fields, recently termed simulation-based inference [36]. In this approach, mechanistic models of the event of interest yield quantitative predictions that are contrasted with data, and the model best explaining the data is kept. We describe recent work that suggests that such a strategy may now be feasible for the question of why the human brain size evolved. We discuss results of this work so far, which find that, counter-intuitively, the human brain size could be an evolutionary by-product of selection for another trait despite the model matching a wide array of observations. We assess whether or not these findings are consistent with available data from fossil and living hominins. In addition, we briefly discuss potential risks of this strategy and possible ways to mitigate these risks.

## 2. A way forward: simulation-based inference

Inferring why the human brain size evolved is particularly challenging because it asks about the causes of a unique event for which natural and artificial experiments are largely impossible. Despite this difficult combination, analogous challenges have been successfully tackled in multiple fields, with *in silico* experiments. This strategy has been recently called simulation-based inference [36] and has played a pivotal role in many scientific domains, including in establishing that humans are causing climate change, in many confirmations of general relativity, in the discovery of the Higgs boson, in epidemiology including in COVID research, and in economics, human population genetics, protein folding and ecology [36].

In general, simulation-based inference involves the following steps: (i) consider observed data generated by the process one seeks to understand; (ii) formulate mechanistic mathematical or computational models of the process and draw predicted data from the models; (iii) test the predicted data against the observed data; and (iv) keep the model(s) that best explain the observed data using statistical inference techniques. The observed data used in this approach need not be experimental and can be observational, and the analysis is not correlational but mechanistic. Because the models are mechanistic where mechanisms are explicitly modelled and can be used to implement *in silico* interventions, the models can give causal explanations for the *in silico* data. The identified *in silico* causes give inferred causes for the observed data, where this inference can be improved by methods that compare the predictions of multiple mechanistic models and reject those models that are less able to explain the data [36–38].

We suggest that simulation-based inference offers a promising way forward to infer why the human brain size evolved. We thus propose a strategy involving the four tailored steps: (i) consider the observed data to be the evolutionary trajectories of brain and body sizes over hominin evolution and the developmental trajectories of brain and body sizes for various hominin species from birth to adulthood. This choice is intended to facilitate model identifiability: two models may yield indistinguishable predictions for evolutionary trajectories but distinguishable predictions for developmental trajectories, facilitating model choice. (ii) Formulate mechanistic mathematical models that yield quantitative predictions for the development and evolution of hominin brain and body sizes. (iii) Test the predicted evolutionary and developmental trajectories of hominin brain and body sizes against observed trajectories. (iv) Use statistical techniques, such as approximate Bayesian computation, for model selection. We discuss below how recent work has taken the first three steps of this strategy and has made the fourth feasible.

## 3. A simulator: the brain model

For simulation-based inference of why the human brain size evolved, we first need a simulator, that is, a mechanistic model that can replicate to some extent the observed evolutionary and developmental trajectories of hominin brain size. One model that does this, termed the brain model, has recently become available [39–41]. In this section, we conceptually describe this model.

**Figure 1.**
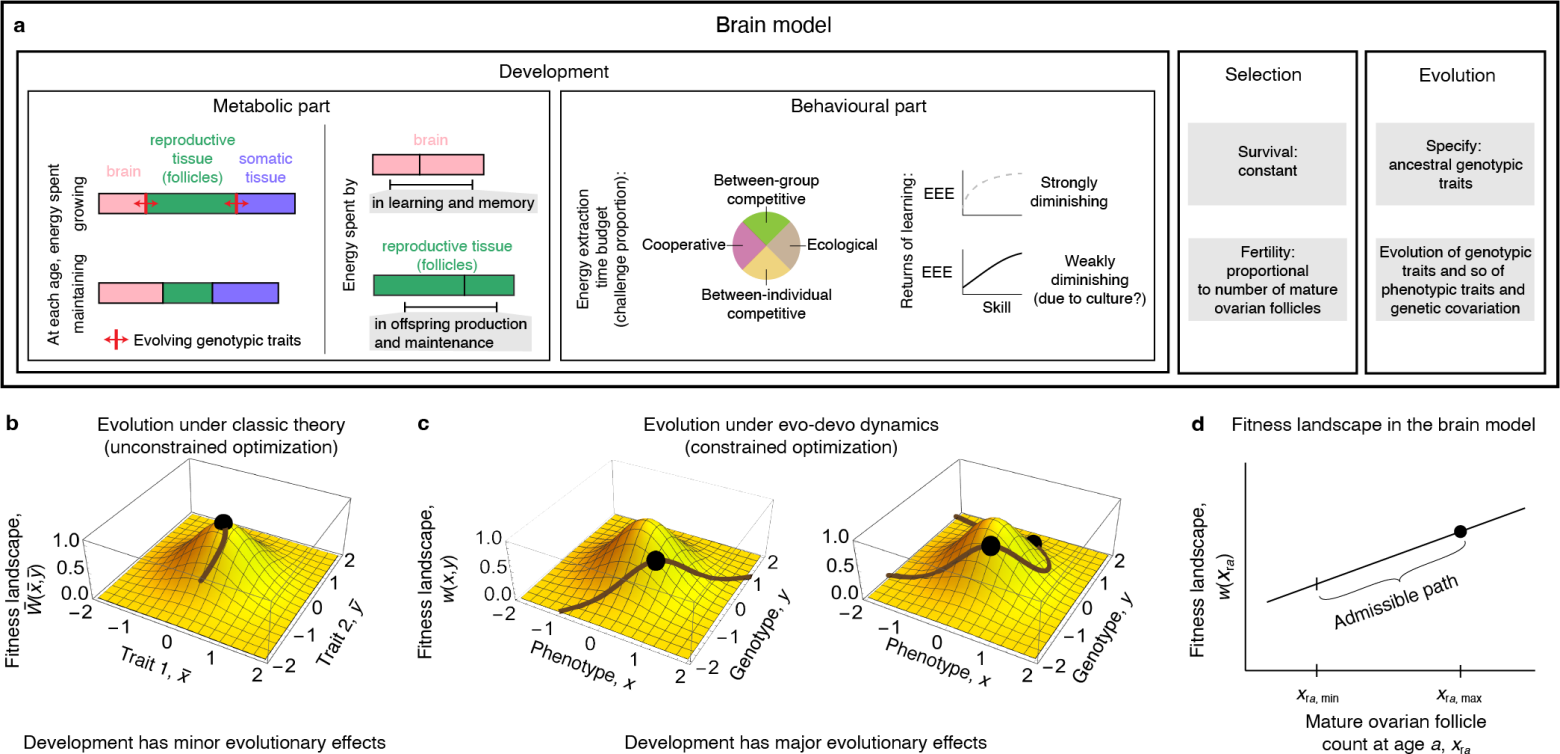
The brain model and evo–devo dynamics theory. (a) Components of the brain model. EEE, energy extraction efficiency. (b) Evolution by natural selection under classic evolutionary theory. Evolution converges to a local fitness peak (dot) [42]. In this view, development diverts the evolutionary trajectory (line) from the direction of steepest fitness increase by generating genetic correlations, but the outcome is a fitness peak regardless; thus, changing development does not change the outcome (dot) in a single-peak fitness landscape, so development has minor evolutionary effects. (c) Evolution by natural selection under evo–devo dynamics theory [43]. The bottom axes cannot be any two traits but must be the phenotypes under consideration and their underlying genotypic traits. The phenotype and the genotype are related by development, so there is genetic variation exclusively along the path (brown line) where the relationship between the phenotype and genotype holds. Evolution is constrained to occur along the path and stops at path peaks rather than landscape peaks. Thus, changing development alone (the path) can change the outcome without change in selection (the landscape). (d) Fitness landscape in the brain model, which only depends on the pre-ovulatory ovarian follicle count $x_{ra}$ at all ages a. Only one axis for a given age a is shown. The slope of the fitness landscape is smaller for axes of increasing age owing to the decreasing force of selection with age. In (b) evolutionary change is $\Delta \bar{\text{z}}={\bar{W}}^{-1}\text{G}\left(\partial \bar{W}/\partial \bar{\text{z}}\right)$ [42], where the overbars denote population-average values, the column vector of average trait values is $\bar{z}=(\bar{x},\bar{y}{)}^{⊺}$, T denotes matrix transpose, W is absolute fitness, the additive genetic covariance matrix is G=(1,0.5;0.5,1) and the semicolon denotes a line break. Thus, **G** is non-singular as is typically assumed, so there is genetic variation in all directions of trait space. In (c), evolutionary change is $\text{d}\bar{\text{z}}/\text{d}\tau =\text{H}\left(\partial w/\partial \text{z}\right)$, where the vector of each individual's trait values is $\text{z}=(x,y{)}^{⊺}$, w is relative fitness, its derivative is evaluated at average trait values, τ is evolutionary time, and the mechanistic additive genetic covariance matrix is $\text{H}=((\text{d}x/\text{d}y{)}^{2},\text{d}x/\text{d}y;\text{d}x/\text{d}y,1)$, so it is always singular, and then there is genetic variation only along the developmental constraint (path); the developmental constraint is x=1+y (left) or $x=1+y+3y^{2}/2$ (right). (c) is taken from [44]. (d) is taken from [41].

The brain model can be seen as having three major components: development, selection and evolution (figure 1a). Development is not typically modelled explicitly in mathematical evolutionary models, except in life history models that assume evolutionary equilibrium [45–48]. The brain model explicitly models development to make quantitative predictions of brain size evolution (i.e. in kilograms) while incorporating empirical data on brain metabolic costs, which are thought to be a key factor limiting the evolution of large brains [49,50]. So the brain model was first formulated as a life history model assuming evolutionary equilibrium [39,40]. Subsequent mathematical theory integrating development and evolution [43] allowed the brain model to predict evolutionary trajectories rather than only evolutionary endpoints [41].

### (a) Development

The development component of the brain model can be seen as involving two parts: a metabolic part and a behavioural part.

#### (i) Metabolic part

The metabolic part specifies the functions of an individual’s tissues and is derived by considering energy conservation. The brain model considers a population of females that can have different ages and reproduce clonally for simplicity. Each female has a body formed by brain tissue, reproductive tissue and the remainder called somatic tissue. The energy that each tissue consumes at every time is assumed to equal the energy spent by the tissue on its growth and maintenance. This assumption follows West *et al*.’s metabolic model of body size development [51] that depends on the metabolic costs of body growth and maintenance, which are costs that are easily estimated from empirical data (from those estimates, West *et al*.’s model correctly predicts the development of body size in many species, but has been criticized for assuming that reproduction is proportional to body size [52], which the brain model does not assume). This makes the brain model depend on parameter values such as brain metabolic costs, which are entered into the model directly from available empirical estimates [53].

Subsequently, the model makes a key assumption that assigns functions to tissues, such that from energy conservation, some of the energy that a tissue consumes at a given time is due to a function the tissue has. Specifically, regarding brain’s function, the model assumes that some of the energy that the brain consumes at a given time is due to the acquisition and maintenance of skills of some type, that is, due to learning and memory of such skills. Similarly, regarding reproductive tissue’s function, the model assumes that some of the energy that the reproductive tissue consumes at a given time is due to the production and maintenance of offspring. Somatic tissue is not assumed to have a specific function, but it contributes to body size, which gives somatic tissue an implicit function as follows.

The energy consumption of an individual at rest is her resting metabolic rate and this describes her energy budget. Also following West *et al*.’s model [51], the brain model assumes that resting metabolic rate relates to body size by a power law, called Kleiber’s law [54], as roughly observed empirically for ontogenetic data (fig. C of [39]). This gives somatic tissue an implicit function by contributing to the energy budget, since all tissues including somatic tissue contribute to resting metabolic rate by contributing to body size. The model assumes standard life history trade-offs [55] such that, out of the energy spent in growth, that allocated to growing a given tissue at a given time is unavailable to grow other tissues.

The key output of the metabolic part is four dynamic equations that describe the development of the three tissues and of skill level, that is, the tissue growth rates and the learning rate. From the learning rate equation it follows that, if skill level plateaus over development, then adult skill is proportional to adult brain mass. This proportionality between adult brain size and adult skill level is more general than other results of the model as it only requires three assumptions: that some of a tissue’s energy expenditure is due to some of the tissue’s function, that some of the brain’s functions are learning and memory, and that a tissue’s energy expenditure is due to the tissue’s growth and maintenance.

#### (ii) Behavioural part

The behavioural part of the model specifies the function of skills. This part is phenomenological in that the mechanisms are not explicitly described by dynamic equations but by equations describing the possible outcome of such dynamics. The behavioural part assumes that the skills are for energy extraction from the environment. Thus, the skills are assumed to affect the individual’s energy budget, specifically, by letting the ‘intercept’ in Kleiber’s law be proportional to a quantity called the individual’s energy extraction efficiency (EEE, which ranges from 0 to 1), which depends on the individual’s skills and those of social partners.

Individuals can obtain energy by solving (figure 1a, behavioural part): ecological challenges where the individual’s skills are pitted against the environment (e.g. foraging or processing food alone), cooperative challenges where both the individual’s skills and those of another individual of the same age are pitted against the environment (e.g. cooperative foraging or food processing), between-individual competitive challenges where the individual’s skills are pitted against those of another individual of the same age (e.g. social manipulation), or between-group competitive challenges where both the individual’s skills and those of another individual of the same age are pitted against those of another pair of individuals of the same age (e.g. group competition). At any time, an individual faces a given proportion of these challenges, which is the energy extraction time budget and is assumed constant over life.

The skills are allowed to affect the individual’s energy extraction efficiency via either a power or exponential function. For the particular parameter values involved in results below, these functions respectively yield an energy extraction efficiency that plateaus quickly or slowly as the individual’s skill increases, that is, they involve strongly or weakly diminishing returns of learning (figure 1a, behavioural part). Maternal care is modelled by increasing with age the extent to which an individual’s energy extraction efficiency depends on her overcoming such challenges.

### (b) Selection

Next, the selection component of the model specifies survival and fertility. This component makes strong assumptions to simplify the mathematical analysis on a first exploration of the model. Yet, despite these strong assumptions, the model was able to recover many observations and so we make them here; these and other assumptions can be relaxed in the future.

The model assumes that the survival probability at every age is constant. It also assumes that competition for resources (i.e. density dependence) affects fertility but not survival, and this keeps the population size at carrying capacity [56], although the carrying capacity may evolve [57,58]. Moreover, the model assumes that reproductive tissue is defined narrowly enough that it is not involved in offspring maintenance, in which case fertility becomes proportional to the size of reproductive tissue. By defining reproductive tissue this narrowly, the metabolic costs of offspring maintenance incurred by the body are then ascribed to the somatic and brain tissues. This simplifying assumption yielded virtually the same results as without it (fig. E of [39]), but allowed further analytical treatment of the original model, which used optimal control.

To operationalize the assumption that reproductive tissue is not involved in offspring maintenance, and as in real females tissues such as the uterus and mammary glands are involved in offspring maintenance during gestation and lactation, reproductive tissue is then defined as referring to pre-ovulatory ovarian follicles, which are follicles at the latest developmental stage before ovulation. This latest stage is taken because ovarian follicles are present from birth in real human females despite being non-reproductive, but follicles at the latest stages of development are only present in reproductively able females [59] and are clinical indicators of fertility [60,61]. Thus, in the model, fertility is proportional to the count of pre-ovulatory ovarian follicles measured in mass units, which is consistent with clinical practice [60,61].

### (c) Evolution

Finally, the evolution component of the model allows it to predict the evolutionary trajectories. Originally, the brain model considered only the end of evolutionary trajectories using optimization by assuming that evolution converged to equilibrium, as is standard in behavioural ecology and life history theory [39,40]. The model then addressed the evolutionary question of how much energy should be allocated to the growth of the different tissues. To do this, the model assumes that the fraction of energy allocated to the growth of each tissue at each age is under genetic control described by so-called genotypic traits (red arrows in figure 1a, metabolic part). An individual’s genotype thus modulates the growth rate of her tissues, whereas an individual’s phenotype is her brain size, body size, follicle count and skill level at each age. At evolutionary equilibrium, these genotypic traits maximise lifetime reproductive success in an evolutionary game theory sense (i.e. they are evolutionarily stable strategies). However, this equilibrium approach meant that evolutionary trajectories could not be predicted. This equilibrium approach had to be taken because of the long-standing limited mathematical integration of development and evolution [62–64], which meant there were no tractable tools to model the dynamics of both for a relatively complex model such as this one.

This problem was overcome by a mathematical theory, termed evo–devo dynamics [43], that integrates development and evolution in a tractable way assuming clonal reproduction and rare, weak and unbiased mutation, as is standard in adaptive dynamics [57,58]. Using evo–devo dynamics rather than optimization, the model then yields predicted evolutionary trajectories [41]. Moreover, evo–devo dynamics provides equations to translate developmentally dynamic equations into genetic covariation, enabling a description of long-term evolution including the evolution of genetic covariation and a separation of the action of selection and genetic constraints on evolutionary change.

Evo–devo dynamics finds that long-term evolution should be understood in a different way from classic theory, which is important to understand the results of the brain model. Specifically, evo–devo dynamics finds that long-term evolution can be understood as the climbing of a fitness landscape by considering the evolution of both phenotypes and their underlying genotypes, rather than only the evolution of phenotypes as in standard quantitative genetics (figure 1b,c) [44]. Then, long-term evolution is constrained to occur along a path on the fitness landscape where the relationship between genotype and phenotype holds, as there is genetic variation only along this path. This entails that evolutionary outcomes occur at peaks of this admissible evolutionary path, not on peaks of the fitness landscape, as traditionally assumed. Hence, development alone (the path) can change evolutionary outcomes without changes in selection (the landscape), even in a single-peak fitness landscape.

Evo–devo dynamics shows that the brain model has a linear fitness landscape that depends only on the pre-ovulatory ovarian follicle count because survival is constant and fertility is proportional to such a follicle count (figure 1d). Thus, brain size is selectively neutral in the model (the slope of fitness is flat with respect to brain size). Moreover, evo–devo dynamics shows that the empirically estimated brain metabolic costs in the model only affect development and so genetic covariation (the path), not selection (the landscape), so they are not direct fitness costs [41].

### (d) Parameters and ancestral conditions

The model output depends on 26 parameters and the initial (i.e. ancestral) conditions, which are the ancestral genotypic traits (parameter values and ancestral genotypic traits are given in the electronic supplementary material). Of the 26 parameters, 12 have values estimated from empirical data, for human females where possible, and they pertain to tissue metabolic costs, tissue sizes at birth, Kleiber’s law parameters, mortality rate and final reproductive age (the model does not yet consider post-reproductive life) [39]; two additional parameters were partly estimated from data either from a closely associated tissue (for the maintenance metabolic cost of mature ovarian follicles) or from an empirically informed range (for the metabolic cost of memory), with their particular value chosen by manual ontogenetic fit (i.e. manually changing the parameter value alone until the evolved phenotypic traits in the model roughly ontogenetically fit the data) (electronic supplementary material, table S1) [40]. A further six parameters have values chosen by hand, set to some reasonable value, and they have virtually no effect on the evolved adult brain and body sizes; these parameters refer to the fraction of brain metabolic rate allocated to skills, skill level at birth, the number of age bins per year and the evolutionary speed (determined by the proportionality factor between fertility and follicle count) (electronic supplementary material, table S2) [39,41]. An additional five parameters have values chosen by manual ontogenetic fit as outlined above and they describe the metabolic cost of learning, maternal care and the returns of learning (electronic supplementary material, table S2) [39,40]. The remaining three parameters specify the energy extraction time budget and have values found by exhaustively searching those that minimize the distance between the observed adult brain and body sizes and the evolved ones in the model (electronic supplementary material, table S2) [40].

**Figure 2.**
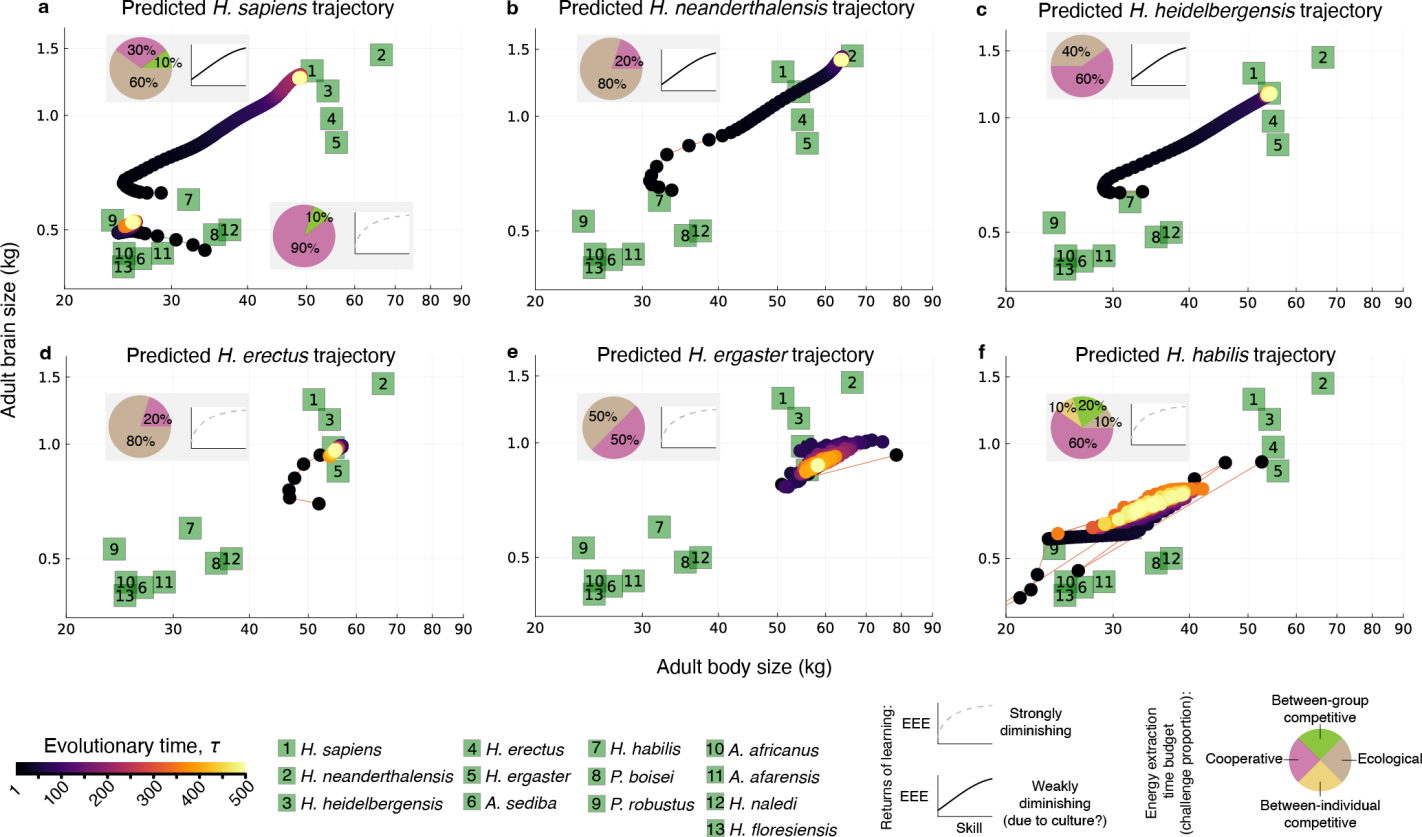
Predicted evolutionary trajectories. Each small black circle shows the predicted adult brain and body sizes at a given evolutionary time. Grey boxes describe the parameter combinations used, which represent energy extraction time budgets and returns of learning. Green squares show the observed adult brain and body sizes in 13 hominin species (data from [53,66–76], using only female data when possible). (a) The bottom trajectory uses parameter values such that starting from a manually chosen initial condition the model converges to brain and body sizes that are of *Australopithecus* scale. The top trajectory starts with the evolved genotype of the bottom trajectory but uses different parameter values, which yields an immediate plastic change in brain and body sizes and the evolution of adult brain and body sizes of *Homo sapiens*. (b–f) Repeating the same procedure of changing parameter values starting from the *Australopithecus*-like genotype (yellow dot in bottom trajectory in (a)) yields the evolution of adult brain and body sizes of *Homo neanderthalensis*, *Homo heidelbergensis*, *Homo erectus*, *Homo ergaster* and *Homo habilis*. (a) Taken with modification from [41]. EEE, energy extraction efficiency. *H, Homo; A, Australopithecus; P, Paranthropus*.

The ancestral conditions were chosen as follows. The predicted developmental trajectories are found to depend on the ancestral conditions [41] (which may be due to the fact that the additive genetic covariance matrix of the phenotype and the genotype is always singular [43,44,65]). To start the model from a realistic ancestral point, the following procedure was taken. The ancestral genotypic traits of the bottom trajectory in figure 2a were chosen by hand (electronic supplementary material, table S3). Their values were chosen to describe decreasing allocation to brain growth over life, and a switch to energy allocation to reproduction at 10 years of age. Subsequently, the model was run under parameter values that yield the evolution of *Australopithecus*-like brain and body sizes (such parameter values were identified by [40] and are described in the bottom grey box of figure 2a, which yields the bottom trajectory in figure 2a; the computer code used to generate all figures is that of [41] and is available online as electronic supplementary material). Organisms with the resulting genotype (at yellow circle in the bottom trajectory in figure 2a) are then exposed to different conditions (top grey box of figure 2a and grey boxes of figure 2b–f), which yields an immediate plastic response in the phenotype (the start of the top trajectory of figure 2a, and of the trajectories in figure 2b–f). Depending on such conditions, subsequent evolution then converges to adult brain and body sizes of *Homo sapiens*, *Homo neanderthalensis*, *Homo heidelbergensis*, *Homo erectus*, *Homo ergaster* and *Homo habilis* (the top trajectory of figure 2a, and the trajectories in figure 2b–f).

Each evolutionary time step (τ) lasts the time it takes for rare mutants to fix. To illustrate how our predictions may translate to real time, we use the yardstick that mutant fixation takes 11.5 kyr (calculated by assuming that mutant fixation takes 500 generations and one generation for females is 23 years [77]). This is only for illustration, as a better approach is to calculate time to fixation from the selection coefficient [78], but we leave that for future work as the parameter ($\eta_{1}$) controlling evolutionary speed in the model has not been calibrated with data.

## 4. Testing predictions against observation

With a simulator in place, the next step for simulation-based inference is to test the model predictions by comparing them with observation. This should be done systematically across many model variations, to maximize the possibility that the model is a suitable description of the real process by maximizing either the likelihood or posterior distribution of the model given the data [36,37]. This systematic comparison with the brain model seems to be now feasible, as the model’s runtime was reduced from a prohibitive 3−4 days per run using optimal control software GPOPS in MATLAB [40,79] to a tractable 3−4 min using evo–devo dynamics in Julia [41,80]. This enhanced computational speed is possibly partly because of the computational speed of Julia and partly because evo–devo dynamics avoids the need of iterated best-response dynamics used to solve the differential game involved in the optimization approach. This systematic comparison would give an inference of why the human brain size evolved with uncertainty quantification. As a first illustration of what this comparison of prediction against data would entail, we here qualitatively test the output of a single model instance against observation, without the systematic and quantitative model comparison involved in simulation-based inference.

The brain model yields a wide range of quantitative predictions, many of which correspond to observed patterns of development and evolution of human brain and body sizes, including several patterns that have been described as puzzling or unique to humans [39–41]. The model has been shown to accurately recover the evolution of adult brain and body sizes for all major species of the genus *Homo* and less accurately for *Australopithecus afarensis* at the final points of the predicted evolutionary trajectories [40,41] (figure 2). The model has also been shown to simultaneously recover the evolution of a long human childhood, a pre-adolescence growth spurt, an adolescence period and an adulthood period, each with mostly correct timing [40,41]. We here report and assess against empirical data additional results along the complete evolutionary trajectories rather than only at their end for conditions that yield the evolution of brain and body sizes of *H. sapiens*, *H. neanderthalensis, H. heidelbergensis*, *H. erectus*, *H. ergaster* and *H. habilis*.

We focus this assessment on three types of predictions and organize the assessment from the most certain based on the fossil evidence to the least certain given available data. These three types of predictions are as follows: (i) evolutionary trajectories of adult brain and body sizes for six hominin species; (ii) developmental trajectories over individuals’ life span for these species; and (iii) two key elements identified by the model as causing hominin brain expansion: time budgets for energy extraction and the effect that learning has on energy extraction efficiency.

### (a) Evolutionary change in adult brain and body sizes

We begin our qualitative testing of the model by comparing adult brain sizes predicted by the brain model [41] with adult brain sizes (proxied by endocranial volumes) observed in the hominin fossil record.

**Figure 3.**
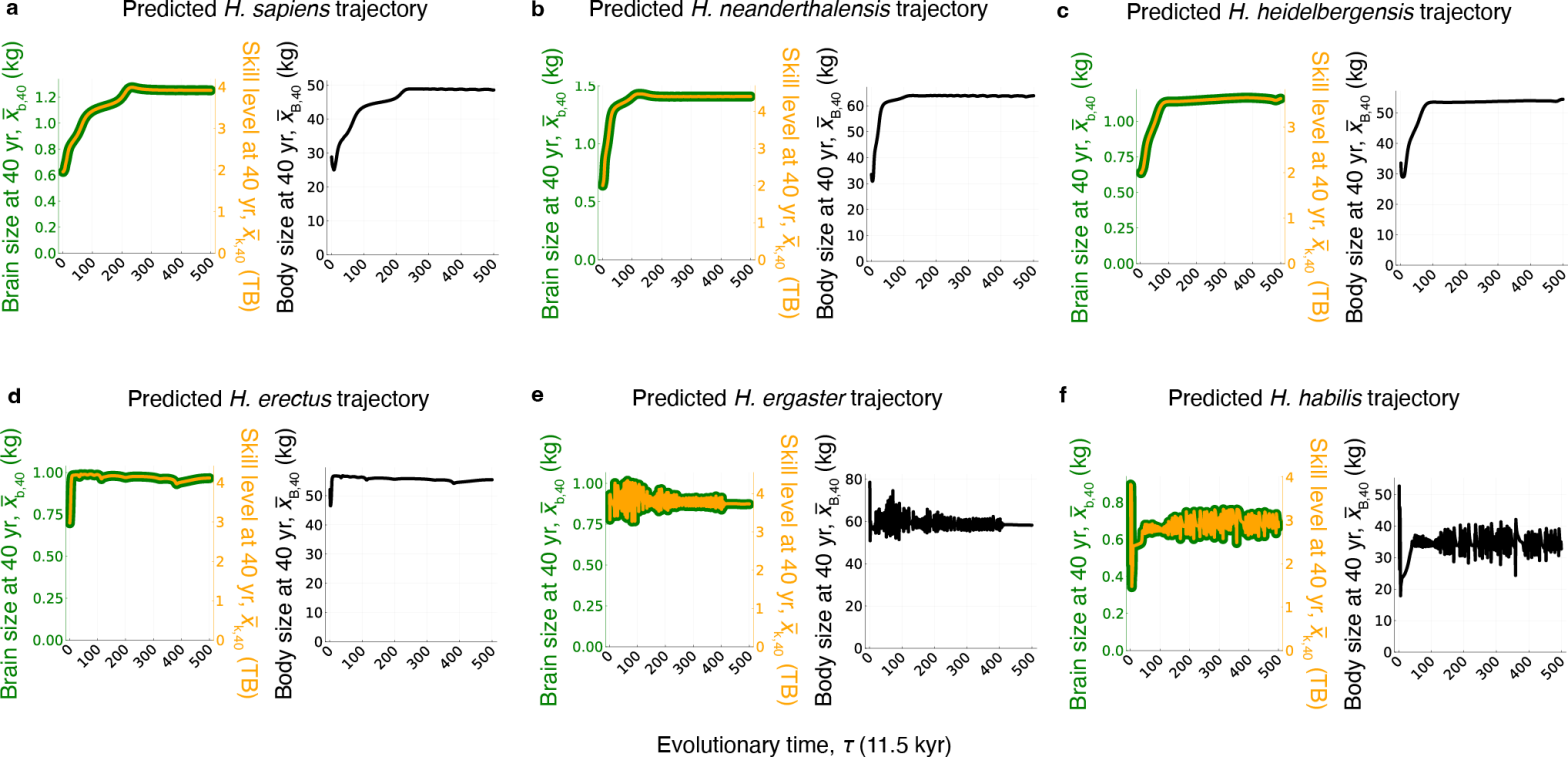
Predicted evolutionary trajectories over time. Plots show the predicted adult brain size, adult skill level and adult body size over evolutionary time corresponding to the trajectories in figure 2. (a) Taken, with modification, from [41]. kg: kilograms. TB: terabytes.

The predicted *H. sapiens* trajectory corresponds relatively well with the values observed in the fossil record (figure 2a). This *H. sapiens* trajectory involves an increase from *H. habilis*-like values to brain and body sizes that are typical of *H. sapiens* over approximately 250 evolutionary steps, which would correspond to the approximately 2.8 Myr from the origin of the genus *Homo* [81] with our yardstick that each evolutionary time step is 11.5 kyr. The *H. sapiens* predicted trajectory recovers to a certain extent the strong increase in brain size attributed to late Pleistocene *H. sapiens* and the subsequent recent decrease in brain size experienced by Holocene modern humans [82] (figure 3a). The brain model shows a brain size decrease (of about 20 g, figure 3a), but this is of a smaller magnitude than that inferred from empirical data [83], as the model does not reach the very large brain sizes typical of late Pleistocene modern humans. The recovered brain size decrease happens earlier than inferred from empirical data (less than 10 ka in empirical data [83], versus approximately 180 ka in the brain model, with a brain size peak at evolutionary step 234 considering step 250 as the present). The late Pleistocene values we have just referred to correspond to mixed-sex samples whereas the brain model refers to females, which may account for some of the discrepancy given the differences in average brain size between males and females [82].

The three predicted evolutionary trajectories corresponding to the latest and largest-brained hominin species (*H. heidelbergensis, H. neanderthalensis* and *H. sapiens*) are relatively in line with what we would expect based on their evolutionary relationships (figure 2a–c). The early part of the three predicted trajectories is similar, starting from roughly similar phenotypic values that would correspond with a *H. habilis*-like ancestor (with predicted brain and body sizes respectively of 625 g and 28.8 kg, 639 g and 33.5 kg, and 639 g and 33.5 kg). In the three trajectories, brain and body sizes increase until they reach those corresponding to each species. For the predicted trajectories of *H. heidelbergensis* and *H. neanderthalensis*, and using our yardstick of 11.5 kyr per evolutionary time step, the model reaches final values exceedingly fast, with the final values for brain and body size attained within the first 100 evolutionary time steps, which would correspond to approximately 1.1 Myr from the beginning of the trajectory at *H. habilis* values (figure 3b,c). This abrupt increase in brain size is not supported by data from the fossil record, as *H. heidelbergensis* typical brain size is not attained until approximately 600 kya [84]. Empirical data indicate that *H. neanderthalensis* shows a fast increase in brain size from a *H. heidelbergensis*-like average brain size of 1241 cm^3^ in the early Neanderthals from Sima de los Huesos [85], dated to approximately 400 ka [86], to values of 1600−1700 cm^3^ in later Neanderthals dated to less than 100 ka, such as those from Le Moustier [87], La Ferrassie [88], Amud [89] or Shanidar [90]. However, as with *H. heildelbergensis*, *H. neanderthalensis* typical brain size values are attained earlier in the brain model than indicated by these empirical data.

The predicted *H. erectus* trajectory starts at brain sizes of approximately 700 g, which is slightly below the range of variation observed in most Asian *H. erectus*, but rapidly stabilizes at values of approximately 1000 g (figure 3d), which are common for this sample [91,92]. Empirical data indicate a slower increase in brain size during the evolution of Asian *H. erectus* [93,94] rather than the rapid change predicted by the brain model.

The predicted evolutionary trajectory for *H. ergaster* is unrealistic, oscillating from very high to very low values, and eventually stabilizing at combinations of brain–body sizes that are consistent with the values observed in this species. This trajectory starts off at relatively high brain and body sizes, caused by a large plastic change induced by the change in parameter values of the model. Both the abrupt change in parameters and the large plastic response of brain and body sizes to change in those parameters are likely unrealistic, the former because parameters may change more gradually in nature than the abrupt parameter change implemented, and the latter because developmental robustness (i.e. canalization) probably limits strong plastic responses in brain and body sizes. Brain sizes for the predicted *H. ergaster* trajectory move between 750 g and more than 1000 g, which is on the high side of the level of variation observed in *H. ergaster* specimens.

Likewise, although the brain model accurately recovers the average adult brain size of *H. habilis*, it does so through an even more unlikely evolutionary trajectory, with brain size oscillating between 400 g and almost 1000 g, possibly also owing to the unrealistically strong plastic response obtained at the starting evolutionary time. A broad range of brain sizes has been suggested for *Homo habilis*, particularly if this species is interpreted *sensu lato* and including *Homo rudolfensis* [95], but the observed range of variation in brain size is from approximately 500 cm^3^ in KNM-ER 1813 [92,96] to approximately 800 cm^3^ in OH 7 [97].

### (b) Species-specific developmental trajectories

We now seek to qualitatively test the predicted developmental trajectories for different hominin species. This test is more uncertain as empirical data on developmental trajectories are substantially more limited than for adult brain sizes. We focus on analysing predictions regarding the age at which adult brain size is attained across species.

**Figure 4.**
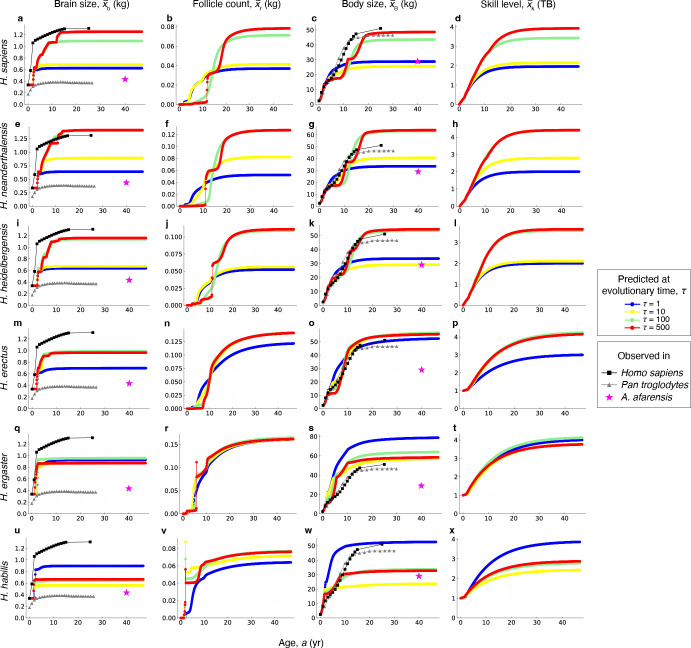
Predicted developmental trajectories. The predicted developmental trajectories underlying the predicted evolutionary are trajectories shown in figure 2. Shown are the developmental trajectories for brain size, pre-ovulatory ovarian follicle count, body size and skill level. The mean observed values in a cross-sectional modern human female sample are shown as black squares (data from tbl. S2 of Kuzawa *et al*. [53], who fitted data from [68]). The mean observed values in cross-sectional *Pan troglodytes* female samples are shown as grey triangles (body size data from fig. 2 of [106]; brain size data from fig. 6 of [98]). The mean observed values in *Australopithecus afarensis* female samples are shown as pink stars (data from tbl. 1 of [73]). For now, the model has used human neonatal brain and body sizes across all trajectories, as shown, but future work may incorporate available estimates for other hominin species [107,108]. (a–d) Taken, with modification, from [41]. *H, Homo; A, Australopithecus.*

Estimates for the age at which adult brain size is attained differ even in present-day species. Classic studies indicate that 90–100% of adult brain size is attained at 5−7 years in humans [98,99], but more recent and extensive magnetic resonance imaging (MRI)-based analyses, which include some longitudinal datasets, indicate that total brain volume peaks at approximately 12 years in humans [100]. Adult brain size is estimated to be attained at between 2 and 5 years in chimpanzees [101,102] and at 3−4 years in mountain gorillas [103]. Given the association between adult brain size and time needed to reach it [104,105], it is expected that earlier and smaller-brained hominin species would have reached adult brain size at a younger age than later and larger-brained hominin species. This expectation is recovered by the brain model, with Neanderthals and modern humans being predicted to attain adult brain sizes beyond 12 years (figure 4a,e), *H. heidelbergensis* around 11 years (figure 4i), *H. erectus* at 7−8 years (figure 4m; see also [109–111]), *H. ergaster* at 3−4 years (figure 4q) and *H. habilis* at 2−3 years (figure 4u).

Starting from *H. heidelbergensis*, the brain model predicts growth trajectories that are stepped with different brain growth spurts, the strongest one being that corresponding to the first year of postnatal life, although later than observed in smoothed cross-sectional curves [98,112,113]. A later and weaker pre-adolescent brain growth spurt is predicted by the brain model before the attainment of adult brain size at 10−12 years, whereas the intermediate childhood period shows a slower rate of growth. These brain growth spurts are not observed in descriptions of aggregated human data, although such descriptions assume smooth trajectories [100], so analyses relaxing this assumption are needed to test the extent to which individual growth curves are smooth or stepped with different spurts. The brain model predicts a gradual evolution to this stepped trajectory (coloured dots and lines in figure 4*a*; supplementary video 1 of [41]), with *H. heidelbergensis* showing an incipient version of the stepped trajectory, and Neanderthals and modern humans showing more clearly the separation between a fast postnatal brain growth, slow childhood brain growth and fast but short pre-adolescent brain growth spurt.

### (c) Species-specific time budgets and returns of learning

The brain model predicts that changes in only two sets of conditions can yield the evolution of brain and body sizes of six major hominin species: the energy extraction time budgets and returns of learning (grey boxes in figure 2). We know of few data to test these predictions against, but the model finds them to be important, so we attempt to discuss their relationship with the limited available data in the hope that future data and analyses can yield stronger assessments.

First, we assess whether the energy extraction time budgets predicted by the brain model correspond to what is known or hypothesized regarding how hominins used their time. The brain model predicts that *H. sapiens* brain and body sizes evolved under a combination of ecological (60%), cooperative (30%) and between-group competitive challenges (10%), and that Neanderthal brain and body sizes evolved under a combination of ecological (80%) and cooperative challenges (20%), where these values were found by fitting predicted and observed adult brain and body sizes [40]. These values predict that *H. sapiens* engaged in cooperative problem-solving twice as much (30% + 10% = 40% of their time) as Neanderthals (20%). Sensitivity analyses show that the larger Neanderthal brain sizes evolve in the model because of engaging in more individual problem-solving (80%, rather than 60% for *H. sapiens* brain sizes) [40]. Although we know of no empirical data to compare these predictions to, they seem consistent with evidence indicating that modern humans engaged more in building complex social networks [114,115].

The brain model predicts that the life of earlier hominin species was dominated by cooperative interactions to obtain food, and in later hominin species life became dominated by individual energy acquisition [40]. This seems to contrast with observation, for instance as cooperative feeding is variable in chimpanzees, with cooperative male hunting being more prevalent in Taï than in Gombe [116], and female cooperative foraging involving smaller groups than in males [117]. Yet, it has been suggested that cooperative breeding was required for the genus *Homo* to evolve a large brain size [118]. This suggestion may be consistent with the brain model in that substantive maternal or allomaternal care is needed for human-size brain evolution over ontogeny in the model (fig. G of [39]) and that the model predicts cooperative foraging in early hominins (figure 2). However, peer cooperation is predicted by the model to cause the evolution of smaller brains because individuals can rely on others’ skills rather than only on their own skills [40], in accordance with previous suggestions [119] (but see [120]).

The model predicts that ecological challenges were more prevalent in the two Eurasian hominin species, Asian *H. erectus* and *H. neanderthalensis*, involving 80% of their energy extraction time budgets. It seems feasible that Eurasian environments, with strong seasonality, involved more prevalent ecological challenges, which the model predicts to cause brain expansion, in accordance with previous suggestions [15,121,122].

Second, we assess the other key factor causing human brain expansion as identified by the model, namely the returns of learning, or the deceleration of energy extraction efficiency as skill level increases. All the model conditions reported above involve diminishing returns of learning: when an individual has low skill level, energy extraction efficiency increases substantially when she increases her skill level, but when an individual has high skill level, energy extraction efficiency does not increase so strongly when she increases her skill level. Under some of the reported conditions, learning returns are either strongly or weakly diminishing: with the latter, the rate at which an individual’s energy extraction efficiency increases when she increases her skill can be sustained as she learns, rather than decaying as with the former. In principle, weakly diminishing returns of learning could arise from culture if individuals can keep learning from accumulated knowledge in the population by enabling further increases in energy extraction efficiency in skilled individuals [40,41]. The brain model predicts a shift from strongly to weakly diminishing returns of learning, that is, to what could be cumulative culture, after the evolution of *H. erectus*, and before the evolution of *H. heidelbergensis*, meaning that *H. heidelbergensis*, *H. neanderthalensis* and *H. sapiens* are the only species considered that would be predicted to have, seemingly, cumulative culture. This agrees with observation. Analyses of stone tool complexity suggest that cumulative culture arose in Middle Pleistocene hominins [123], which would make cumulative culture part of the behavioural repertoire of *H. heidelbergensis, H. neanderthalensis* and *H. sapiens*. Similarly, other complex behaviours that would be indicative of cumulative culture, such as fire control and woodworking, are also thought to have evolved or consolidated during the Middle Pleistocene [124,125]. Yet, the treatment of culture remains implicit in the model, and a more explicit consideration of culture is needed before the model can make more decisive predictions in this regard.

## 5. Why does the human brain size evolve in the model?

Given that the brain model recovers many, although not all, patterns of human brain size evolution and development, we can use it to address our question of why the human brain size evolved, at least in this imperfect *in silico* replica. Sensitivity analyses, which are *in silico* interventions, show that, in the model, two key factors causing brain expansion from australopith to *H. sapiens* sizes are: experiencing a challenging ecology and cumulative culture, specifically, facing a larger proportion of ecological challenges in the energy extraction time budget, and transitioning from strongly to weakly diminishing returns of learning; by contrast, neither cooperation nor competition causes the predicted human brain expansion in this model [40]. One would be inclined to interpret this as ecology and culture increasing selection for brain size, but this is not necessarily correct.

### (a) Direct and total selection in the model

Evo–devo dynamics [43] provides tools to analyse why human brain sizes evolve in the brain model, including to establish what is under selection. This involves an important distinction between direct and total selection. Direct selection is what selection directly acts on; for instance, direct selection for milk production or fruit yield in artificial selection by animal or plant breeders, described by Lande’s selection gradient [42]. In turn, total selection includes direct and indirect selection, described by total selection gradients [43,126–129]. For instance, if a behaviour at a given age increases body size at a later age and body size at that age is under direct selection, then there is indirect and so total selection for the behaviour, even if there is no direct selection for it. In real organisms, what is under direct selection is typically unknown except in artificial selection. Yet, in simulated organisms as we have here, direct selection can be known.

Evo–devo dynamics shows that there is no direct selection for brain size or skill level in the model but only for pre-ovulatory ovarian follicle count (figure 1*d*) [41]. This is necessarily the case given that survival is constant and fertility only depends directly on follicle count (§3b). Although perhaps trivial in hindsight, this observation was not evident in the life history version of the model because there were no tools to write it with a separation of direct selection and genetic constraints [39]. Moreover, evo-dynamics shows that brain size, body size, pre-ovulatory ovarian follicle count, and skill level at any age evolve in the model only if they are genetically correlated with pre-ovulatory ovarian follicle count at reproductively mature ages. Furthermore, evo–devo dynamics shows that the effects of ecology and culture in the model are not to increase direct selection for brain size or any other trait as traditionally assumed, but to change development and so the genetic covariation, including between brain size and pre-ovulatory ovarian follicles. That is, ecology and culture cause brain expansion in the model by affecting the admissible evolutionary path on the fitness landscape, which affects path peaks on the fitness landscape, without affecting the fitness landscape (figure 1*c*,*d*) [41].

Therefore, by contrast with standard thinking, all the evolutionary patterns recovered above, including the evolution of brain and body sizes from *H. habilis* to *H. sapiens* scale, a long childhood, a pre-adolescent growth spurt and an adolescence period are by-products of direct selection for having more pre-ovulatory ovarian follicles. The different conditions (energy extraction time budgets and returns of learning) that yield the evolution of brain and body sizes of six major hominin species in figure 2 exclusively affect development and so genetic covariation, not direct selection. In particular, the human brain size evolves in the model because ecology and culture make brain size genetically correlated with pre-ovulatory ovarian follicles [41].

This positive genetic correlation means that mutations in the genotype that increase brain size also tend to increase follicle count, or mutations that decrease one also tend to decrease the other (pleiotropy). This genetic correlation does not mean that brain size or skill level are fully determined by genes in the model, which they are not. Instead, such genetic correlation depends on development and anything that influences it, including ecology and culture, as development determines the effects of genotype on brain size and follicle count. Unfortunately, the term genetic covariation is misleading because it suggests that it depends on genetics alone, but it depends on development and anything that influences it, so it is far from being only genetically determined. The genetic correlation that causes brain expansion is not necessarily present in the model; for instance, it is absent if the ancestral genotypic traits are such that ancestral individuals develop a small body size, which causes brain size collapse over evolution, necessarily because brain size and pre-ovulatory ovarian follicles become negatively genetically correlated (fig. S1h of [41]).

The genetic correlation that causes brain expansion arises under certain conditions that affect development, including facing a challenging ecology and cumulative culture and having an ancestor with large body size, which affect the individual’s energy budget and so her development, in turn affecting the genetic covariation between brain size and follicle count from the ancestral state onwards. If one implements an *in silico* intervention where individuals both face more ecological challenges and switch from strongly to weakly diminishing returns of learning, an intervention that is mathematically proven in the model not to change direct selection but the genetic covariation between brain size and pre-ovulatory ovarian follicles, then brain and body sizes in the model evolve from australopith scale to *H. sapiens* scale [41] (figure 2*a*). Thus, in the model, the human brain size evolves because a challenging ecology and cumulative culture affect development, making pre-ovulatory ovarian follicles and brain size genetically correlated.

### (b) The human brain size is a spandrel in the model

These findings mean that the human brain size in the model matches Gould & Lewontin’s notion of a spandrel [130], defined as a ‘by-product’ (their word) of direct selection for something else. This definition does not entail that spandrels are maladaptive or non-functional; in the architectural illustration of Gould & Lewontin, spandrels are neither maladaptive nor non-functional as they contribute to sustain the cathedral’s upper structure. In the model, mature ovarian follicle count is the ‘arch’, which in our evolutionary context refers to what is under direct selection (figure 1*e*); instead, hominin brain expansion, the brain and body sizes of six hominin species, a long childhood, a pre-adolescent growth spurt, and an adolescence period are all here by-products of direct selection for pre-ovulatory ovarian follicles.

Evo–devo dynamics also provides tools to assess whether human-sized brains in the model are maladaptive by computing their total effect on fitness. Doing so shows that brain sizes over the course of the recovered human brain expansion are ancestrally adaptive but become slightly maladaptive: total selection for them is typically positive during early evolutionary times (with *H. habilis* brain and body sizes), and becomes typically slightly negative during late evolutionary times (with *H. sapiens* brain and body sizes; Extended Data fig. 3a of [41]). This might help explain the evolved slight reduction in brain size observed in recent *H. sapiens,* although establishing this requires further analyses. Despite being spandrels in the model, human-sized brains are functional by enabling learning and memory of energy extraction skills (§3).

Also, by contrast with long-standing thinking, the human brain size in the model is not an adaptation under a trait-level definition of adaptation. Multiple interpretations of adaptation exist [131], but a trait-level definition of adaptation is ‘a characteristic that enhances the survival or reproduction of organisms that bear it, relative to alternative character states’ [132, p. 56]. This definition admits various interpretations: for instance, as referring to direct selection or total selection. Further elaborations of this definition indicate that it refers to direct selection: ‘Not all traits are adaptations. There are [...] other possible explanations [...]. [T]he feature may have evolved not because it conferred an adaptive advantage, but because it was correlated with another feature that did.’ [132, pp. 67−68]. Interpreting this definition as referring to direct selection, then the human brain size is not an adaptation in the model, since brain size does not directly affect survival or reproduction in the model. Instead, interpreting this definition as referring to total selection, then the human brain size in the model still does not strictly meet the definition, as slightly smaller brains would indirectly increase fitness (red dots in Extended Data fig. 3a of [41]), but such reduced brains do not evolve. The reason is that genes do not directly control brain size in the model, so evolution stops with persistent total selection for brain size decrease, since evolutionary equilibria occur when total *genotypic* selection vanishes, not when total phenotypic selection vanishes (eqs (4) and (6), and unnumbered equation before §S3.3, all of [41]).

However, the evolved individuals with human-sized brains are adapted under an organism-level definition of adaptation. Evo–devo dynamics suggests an organism-level notion of adaptation defined as the process of fitness increase for individuals with average phenotypic and genotypic traits, where, for this model’s assumptions, individuals are adapted if they have *genotypic* traits that totally enhance their fitness relative to other genotypic traits, given the constraints and ancestral genotypic traits. In that sense, the individuals with human-sized brains that evolve in the model are nearly adapted as total genotypic selection becomes nearly zero (red dots in Extended Data fig. 3e–g of [41]) and will become fully adapted given enough time even if total selection for brain size decrease persists.

### (c) Counter-intuitive insights from integrating development and evolution

It can also be said that brain expansion in the model is counter-intuitively caused by developmental constraints defined as the rules of phenotype construction imposed by the dynamic equations describing development. Developmental constraints so defined are dynamic constraints as defined in optimal control theory, give the admissible evolutionary path on the fitness landscape, and can yield ‘biases on the production of variant phenotypes or limitations on phenotypic variability caused by the structure, character, composition or dynamics of the developmental system’ [133]. The developmental constraints that occur in the model when individuals experience a challenging ecology and seemingly cumulative culture make brain and follicles genetically correlated, triggering the evolution from *Australopithecus* to *H. sapiens* brain and body sizes. Without such developmental constraints, brain expansion is not directly favoured in the model and human brain sizes do not evolve, at least for the conditions evaluated so far; instead, with such constraints, brain expansion is still not directly favoured but evolves as a result of the genetic correlations generated. Thus, unexpectedly to us, but as anticipated by Gould & Lewontin, and in agreement with proponents of an extended evolutionary synthesis [134–137], ’constraints themselves become more interesting and more important in delimiting pathways of change than the selective force that may mediate change when it occurs’ [130].

These conclusions are non-standard, but they emerge from our integrated consideration of developmental and evolutionary dynamics, which had remained prohibitive. The absence of direct selection for brain size or skill level in the model contrasts with the longstanding view that human brain expansion was caused by direct selection for increased cognitive ability or behavioural complexity that increases survival, as this is not the case in the model, but such selection may be introduced in the model in the future. Finlay and colleagues have previously suggested that the human brain size could be a spandrel, although for different reasons based on correlational analyses, a suggestion that was generally dismissed by their commentators essentially based on the intuition that an extremely adaptive, complex, outlying and costly trait is unlikely to be a spandrel [138], an intuition that the brain model finds to be incorrect.

Supporting these results, a growing body of empirical evidence has found a genetic association between brain-related traits and fertility-determinant traits in human females [139] and males [140]. For instance, genes associated with skull shape are more highly expressed in female reproductive systems [139], there is a large overlap in the genes expressed in the brain and testis [141], and macrocephaly can occur through mutations involved in male reproduction [140,142].

## 6. Conclusion

We have outlined a strategy to advance our understanding of why the human brain size evolved. The strategy involves formulating mechanistic models that predict evolutionary and developmental trajectories, and model comparison to determine which model best explains the data. The strategy rests on the assumption that, even if all models are wrong, some may be less wrong and they may be identified by comparing their predictions to observations. Difficulties may arise when deciding which models best explain the data, particularly if different models make the same predictions (lack of model identifiability; e.g. [143, pp. 183−207; 127, pp. 253−255; 52]). These difficulties could be mitigated by ‘mak[ing] theories elaborate’ as recommended by Fisher in a meeting discussion [33,144, p. 252] such that models make wide-ranging predictions as the brain model does, which may diminish the possibility that the least wrong models are equally wrong. We have begun the illustration of this strategy for the question of why the human brain size evolved by qualitatively testing a model’s predicted evolutionary and developmental trajectories with available data, but quantitative testing is possible and there are rapidly advancing tools for this task [36]. Although there is uncertainty in the data used, these data contain valuable information that this approach seeks to make the most use of including by pointing to key data gaps to target for improved estimates. Being a single model so far, the brain model has only offered a relatively uncertain answer for why the human brain size evolved, namely as a by-product of selection for fertility-determinant traits, even though it is consistent with recent empirical research [139–142]. The approach proposed here allows advance towards more certain answers, by contrasting more models or model variations against data. Recent mathematical tools discussed here seem to make this strategy feasible.

## Data Availability

The computer code used to generate all the figures is that of [41], modified slightly to generate figures reported here. Supplementary material is available online [145].
